# Pregnancy risk factors associated with birthweight of infants born to Australian Aboriginal women in an urban setting - a retrospective cohort study

**DOI:** 10.1186/s12884-018-1946-3

**Published:** 2018-09-24

**Authors:** Elisa J. Ford, Thomas J. Cade, Lex W. Doyle, Mark P. Umstad

**Affiliations:** 1 Division of Maternity Services, The Royal Women’s Hospital, 20 Flemington Rd, Parkville, VIC 3052 Australia; 2 Department of Obstetrics and Gynaecology, University of Melbourne, The Royal Women’s Hospital, Parkville, VIC 3052 Australia; 3 Research Office, The Royal Women’s Hospital, Parkville, VIC 3052 Australia; 4 Clinical Sciences, Murdoch Children’s Research Institute, Parkville, VIC 3052 Australia

**Keywords:** Australian Aboriginal, Birthweight, Pregnancy, Health, Urban

## Abstract

**Background:**

A key focus of the Closing the Gap campaign is to reduce low birthweight in Aboriginal babies. Limited research exists on factors affecting Aboriginal birthweight in urban areas.

**Methods:**

Retrospective cohort analysis of 38,382 births (38,167 non-Aboriginal, 215 Aboriginal) at the Royal Women’s Hospital in Melbourne from January 2010 to December 2015. Aboriginal status was defined by mothers who identified themselves and their baby as Aboriginal or Torres Strait Islander. The aim was to examine the association of maternal health risk behaviours and obstetric complications with birthweight of infants born to Australian Aboriginal women birthing in an urban setting.

**Results:**

Aboriginal babies had a lower mean birthweight than non-Aboriginal babies (mean difference -290 g; 95% confidence interval [CI] -413, − 166 g), but when accounting for gestational age and sex there was little difference (mean difference 5 g; 95% CI -53, 6 g). Aboriginal babies were significantly more likely to be delivered preterm < 37 weeks (23.3% vs 7.9%, odds ratio [OR] 3.58; 95% CI 2.58, 4.95) and be of low birthweight < 2500 g (22.3% vs 6.7%, OR 4.03; 95% CI 2.90, 5.60) or very low birthweight < 1500 g (9.8% vs 1.8%, OR 5.81; 95% CI 3.67, 9.16).

Aboriginal mothers were significantly more likely to be teenage mothers (9.8% vs 1.6%, OR 5.72; 95% CI 3.54, 9.24), smoke cigarettes throughout the pregnancy (53.8% vs 5.6%, OR 17.2; 95% CI 12.8, 23.0), and use drugs (26.5% vs 2.4%, OR 14.3; 95% CI 10.4, 19.6) during pregnancy, all of which were associated with lower birthweight. Aboriginal mothers were also more likely to have a mental health diagnosis (49.5% vs 18.8%, OR 3.77; 95% CI 2.86, 4.97), be overweight (59.9% vs 42.6%, OR 1.88; 95% CI 1.39, 2.56) and have diabetes (15.3% vs 7.3%, OR 2.31; 95% CI 1.59, 3.35) which were all associated with higher birthweight.

**Conclusions:**

Aboriginal babies born in metropolitan Melbourne are more likely to be of low birthweight compared with non-Aboriginal babies, which in turn was related to higher rates of prematurity and not to being small for gestational age.

## Background

Reducing the incidence of low birthweight Aboriginal babies has been a focus for the Australian Government’s Closing the Gap campaign [1], which aims to reduce the gap in life expectancy between Aboriginal and non-Aboriginal Australians.

Prior to the commencement of the 2008 Closing the Gap campaign, key determinants of Aboriginal birthweight were identified as malnutrition, remoteness, maternal cigarette smoking, limited antenatal care, genitourinary tract infections, and teenage pregnancy [2–4]. Over recent years Aboriginal lifestyles have been significantly influenced by urbanisation and the effects of government campaigns targeting Aboriginal wellbeing. Between 1986 and 2009, trends were observed of increasing diabetes in pregnancy, decreasing rates of urinary tract infections and teenage pregnancies, and little improvement in smoking rates for Aboriginal pregnant women living in Western Australia [5]. The Aboriginal population have progressively become more urbanized with 34% currently living in major cities, and more growth projected for urban than remote areas [6]. Location of residence affects Aboriginal culture, socio-economic circumstances, genetic admixture and access to services. There has been limited research since the Closing the Gap campaign was initiated that have evaluated how these influences have affected determinants of Aboriginal birthweight.

The aim of this study was to compare the prevalence of modifiable health risk behaviours and pregnancy complications experienced by Aboriginal and non-Aboriginal mothers who gave birth in an urban setting at the Royal Women’s Hospital (RWH) in Melbourne, and to quantify the extent each variable was associated with birthweight.

## Methods

For this cohort study, we included all singleton livebirths at RWH after 20 weeks’ gestation and with birthweights more than 400 g between 1st January 2010 and 31st December 2015.

Maternal Aboriginal or Torres Strait Islander status (hereafter referred to as Aboriginal) was recorded both on hospital admission and after the birth of the baby at which time Aboriginal mothers were also asked if their baby was Aboriginal. These data sets were found to have missing data and were occasionally discordant. Rates of missing data on Aboriginal status improved after 2011 when it became mandatory to enquire about Aboriginal status. Concordance between the data sets was 97% when Aboriginal status was determined by the mother identifying both herself and her baby as Aboriginal. Aboriginal status was considered the primary exposure variable, with birthweight and birthweight z-score (birthweight for gestational age and sex) being the primary outcomes.

Pregnancy factors associated with birthweight were identified from the antenatal record and represented the primary risk variables reported in the literature. These were body mass index (BMI), diabetes, Aboriginal status, antepartum haemorrhage (APH), urinary tract infection, diabetes, mental health diagnoses, hospital of intended birth, and cigarette, alcohol, and drug use. Neonatal variables included Aboriginal status, birthweight, sex and gestational age. Gestational age was determined by menstrual history and confirmed by the earliest pregnancy ultrasound. If hospital of intended birth was not listed as RWH they were deemed an ‘in utero transfer.’ Data were extracted from the hospital’s electronic database and were anonymized on collection. Methods of collecting and recording data were unchanged during the study period.

Smoking, alcohol and drug use relied on maternal self-reporting. Any mental health diagnosis recognized by the Diagnostic and Statistical Manual of Mental Disorders (DSM-5) was recorded if reported by the mother or her general practitioner at her first antenatal appointment, or diagnosed during pregnancy. Diabetes status included diagnoses of type 1, type 2 from either before or during pregnancy, and gestational diabetes. Pre-eclampsia/HELLP syndrome was diagnosed if blood pressure more than 140/90 mmHg was recorded on at least two occasions 6 hours apart, combined with fetal growth restriction or signs of renal, haematological, liver or neurological involvement.

**Fig. 1:**
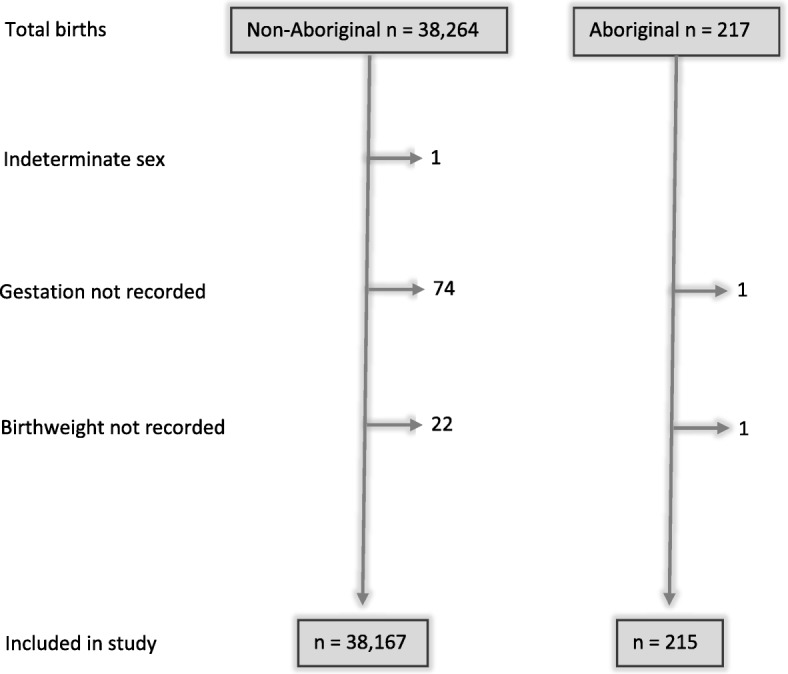
Flow chart demonstrating exclusions from the study population

One baby was excluded due to sex being indeterminate at birth. Records with missing data on BMI, smoking status or mental health diagnosis were excluded (refer to denominators in Table 1). Additionally, one Aboriginal and 74 non-Aboriginal records were excluded due to gestation being unknown, and one Aboriginal and 22 non-Aboriginal records were excluded due to birthweight being missing (Fig. 1).

**Table 1:** Perinatal and sociodemographic data compared between Aboriginal and non-Aboriginal births

Variable	Aboriginal *n* = 215	Non-Aboriginal *n* = 38,167	Statistics	95% CI	*p*-value
In-utero transfer	26 (12.1%)	1345 (3.5%)	3.77^a^	2.49, 5.73	< 0.001
Maternal age (years) – mean (SD)	27.4 (6.7)	30.7 (5.2)	−2.9^b^	−3.7, − 2.0	< 0.001
Maternal age < 20 years	21 (9.8%)	628 (1.6%)	5.72^a^	3.54, 9.24	< 0.001
BMI > 25	115/192 (59.9%)	15,622/36682 (42.6%)	1.88^a^	1.39, 2.56	< 0.001
Diabetes	33 (15.3%)	2802 (7.3%)	2.31^a^	1.59, 3.35	< 0.001
Smoking (any)	123/210 (58.6%)	4070/38036 (10.7%)	10.9^a^	8.16, 14.5	< 0.001
Smoking throughout pregnancy	113/210 (53.8%)	2123/38036 (5.6%)	17.2^a^	12.8, 23.0	< 0.001
Drug use	57 (26.5%)	916 (2.4%)	14.3^a^	10.4, 19.6	< 0.001
Alcohol use	14 (6.5%)	237 (0.6%)	11.1^a^	6.38, 19.4	< 0.001
Mental health diagnosis	106/214 (49.5%)	7185/38144 (18.8%)	3.77^a^	2.86, 4.97	< 0.001
APH	24 (11.2%)	1870 (4.9%)	2.40^a^	1.57, 3.68	< 0.001
Pre-eclampsia/HELLP	9 (4.2%)	791 (2.1%)	2.07^a^	1.06, 4.03	0.033
UTI in pregnancy	2 (0.9%)	188 (0.5%)	1.93^a^	0.48, 7.79	0.36
Hypertension^c^	14 (6.5%)	1369 (3.6%)	1.79^a^	0.98, 3.25	0.058
Male sex	123 (57.2%)	19,681 (51.6%)	1.25^a^	0.96, 1.64	0.09
Birthweight (g) – mean (SD)	3036 (893)	3320 (607)	−290^b^	−413, −166	< 0.001
High birthweight > 4000 g	17 (7.9%)	3763 (9.9%)	0.77^a^	0.46, 1.31	0.34
Low birthweight < 2500 g	48 (22.3%)	2576 (6.7%)	4.03^a^	2.90, 5.60	< 0.001
Very low birthweight < 1500 g	21 (9.8%)	701 (1.8%)	5.81^a^	3.67, 9.16	< 0.001
Gestational age (weeks) – mean (SD)	37.3 (3.9)	38.8 (2.3)	−1.6^b^	−2.1, − 1.1	< 0.001
Preterm < 37 weeks	50 (23.3%)	3002 (7.9%)	3.58^a^	2.58, 4.95	< 0.001
Birthweight z-score – mean (SD)	−0.04 (1.14)	− 0.06 (0.93)	0.03^b^	− 0.13, 0.19	0.72

Data are n (%), unless otherwise specified. Denominators added if missing data

^a^odds ratio; ^b^mean difference; ^c^essential or pregnancy-induced

*CI* Confidence Interval, *BMI* Body Mass Index, *APH* Antepartum Haemorrhage, *HELLP* Haemolysis, Elevated Liver enzymes and low Platelet levels

### Statistical methods

Birthweight z-scores (standard deviation scores) were calculated using the British 1990 growth reference [7] to adjust for sex and gestational age. The British reference was chosen because z-scores for babies of all gestational ages can be calculated down to 23 weeks. Any systematic differences in z-scores between Australian and British populations is not a concern for the current study because we were interested only in the differences between Aboriginal and non-Aboriginal infants within our sample, which would be similar regardless of the reference standard used to calculate z-scores,

SPSS statistical analysis software (version 24.0) and Stata (version 14.2) were used for data analyses. Outcomes were compared between the groups using linear (continuous) and logistic (binary) regression, with models fitted using generalised estimating equations (GEEs), and reported with robust (sandwich) estimates of standard errors to allow for clustering of women who delivered more than once during the study period. Linear regression with GEEs was also used to determine the associations of maternal variables with not only birthweight, but also birthweight z-scores to be able to correct for the effect of gestational age on birthweight.Mean differences with 95% confidence intervals (CIs) or odds ratios (ORs) and 95% CIs were calculated from regression coefficients and their standard errors.

The project was approved as an audit by The Royal Women’s Hospital’s Human Research Ethics Committee on 25th October, 2016.

## Results

This study included a total of 38,382 livebirths, including 38,167 non-Aboriginal and 215 Aboriginal livebirths (Table 1) of which 51.6% were male; 18 (8.3%) Aboriginal women and 5647 (14.8%) non-Aboriginal women had more than one birth over the study period. Aboriginal women were on average 2.9 years younger than non-Aboriginal women when their child was born, and they had more teenage pregnancies.

Aboriginal women were found to have higher levels of potentially modifiable health risk behaviours than non-Aboriginal women and were more likely to have poor nutrition as evidenced by having a BMI > 25. Aboriginal women were significantly more likely to smoke cigarettes, drink alcohol and use drugs than non-Aboriginal women (Table 1). The most common drugs used by Aboriginal pregnant women were cannabis (*n* = 37, 60.3%), followed by methadone (*n* = 23, 39.7%), opioids (*n* = 11, 19.0%), and amphetamines (*n* = 8, 13.8%). Polydrug use was common, with 41.4% of Aboriginal pregnant drug users using two or more drugs. Aboriginal women had more complications of pregnancy than non-Aboriginal women with significantly higher rates of diabetes, antepartum haemorrhage, and pre-eclampsia syndromes.

Aboriginal babies had a gross average birthweight 290 g lower than non-Aboriginal babies (Table 1). They were more likely to be classified as low birthweight and more than five times more likely to be of very low birthweight compared with non-Aboriginal babies. Aboriginal babies were born on average 1.6 weeks earlier than non-Aboriginal babies, and were three times more likely to be preterm. There was little difference in birthweight z-scores between Aboriginal and non-Aboriginal babies in a univariable analysis (Table 1).

In the multivariable analyses, being a teenage mother, smoking throughout the pregnancy, drug use, antepartum haemorrhage, pre-eclampsia and being a multiple birth were all associated with lower birthweight, whereas BMI > 25, diabetes, having a mental health diagnosis, male sex, and increased maturity were all associated with higher birthweight. After adjustment for all the perinatal variables, the difference in birthweight between Aboriginal and non-Aboriginal babies diminished,(Table 2).

**Table 2:** Multivariable linear regression analysis for variables associated with a) birthweight and b) birthweight z-score – all variables are adjusted for the presence of all others

	a) birthweight (g)			a) birthweight z-score		
	Mean difference ^a^	95% CI	*p*-value	Mean difference ^a^	95% CI	*p*-value
Aboriginal status	44	−22, 110	0.19	0.102	− 0.050, 0.254	0.19
In-utero transfer	−74	−102, −45	< 0.001	−0.173	− 0.244, − 0.102	< 0.001
Maternal age < 20	−82	−117, −46	< 0.001	−0.185	− 0.264, − 0.106	< 0.001
BMI > 25	122	113, 131	< 0.001	0.266	0.247, 0.286	< 0.001
Smoking throughout pregnancy	−104	−123, −85	< 0.001	−0.222	− 0.265, − 0.179	< 0.001
Alcohol use	−37	− 93, 19	0.20	− 0.064	− 0.192, 0.063	0.32
Drug use	−93	− 123, − 63	< 0.001	− 0.210	− 0.279, − 0.140	< 0.001
Mental health diagnosis	51	40, 62	< 0.001	0.113	0.088, 0.137	< 0.001
APH > 20/40	−31	−49, − 13	0.001	− 0.040	− 0.082, 0.001	0.057
UTI in pregnancy	1	−60, 61	0.98	0.004	−0.131, 0.139	0.95
Hypertension^b^	−25	−50, −1	0.042	−0.054	−0.111, 0.002	0.06
Diabetes	50	31, 69	< 0.001	0.176	0.132, 0.219	< 0.001
Pre-eclampsia/HELLP	−213	− 248, −178	< 0.001	−0.543	−0.629, − 0.458	< 0.001
Male sex	137	129, 145	< 0.001	−0.010	−0.028, 0.008	0.28
Gestational age (per week)	182	180, 184	< 0.001	−0.019	−0.025, − 0.014	< 0.001

^a^for one unit change in independent variable; ^b^essential or pregnancy-induced

The same variables as for birthweight were also associated with birthweight z-scores, with the exception of antepartum haemorrhage and sex of the infant; the latter because sex is accounted for in calculating birthweight z-scores (Table 2). Moreover, the association of birthweight z-score with gestational age was negative, and not positive as it was for birthweight. After adjustment for all the perinatal variables, the birthweight z-score was marginally higher in Aboriginal than non-Aboriginal babies.

## Discussion

The major finding of this study was that Aboriginal babies weighed less than non-Aboriginal babies born at a large urban maternity centre, but the difference was related to immaturity, rather than to being small for gestational age. Aboriginal babies were three times more likely than non-Aboriginal babies to be born preterm and to be of low birthweight. Aboriginal mothers compared with non-Aboriginal mothers were younger, and they had higher rates of health risk behaviours, including smoking and drug and alcohol use, and of medical complications, including diabetes, overweight, mental health problems, pre-eclampsia, and hypertension. When adjusted for the perinatal differences between the groups, the birthweight and the birthweight z-score of Aboriginal babies were similar to non-Aboriginal babies.

Our finding that Aboriginal babies have similar fetal growth characteristics compared with non-Aboriginal babies is consistent with previous studies. A study of 810 Aboriginal babies born in Cairns found more preterm births but no significant difference in birthweight when accounting for gestational age [8], and another Australian study found no difference in growth using serial ultrasound prior to 20 weeks gestation [9].

Early studies viewed birthweight as a reliable indicator of Aboriginal neonatal health because it related to mortality risk and considered both intrauterine growth restriction and preterm birth [2, 10]. It was suggested that Aboriginal women were shorter and had smaller babies than non-Aboriginal women but with less mortality at low birthweights [4, 11]. Pure-descent Aboriginal babies were found to be lighter than babies with non-Aboriginal admixture but disagreement existed over whether this was due to preterm delivery alone or also due to fetal growth restriction [3, 11]. Limitations of early studies on Aboriginal health outcomes included small sample sizes, difficulties with determining gestational age, missing data, and minimal focus on urban areas [4, 10, 12]. Recent studies agree that preterm birth is the main driver of birthweight disparity [8, 9, 12] and gains in Aboriginal health would be better assessed using gestational age rather than birthweight.

The difference in average birthweight of − 290 g between Aboriginal and non-Aboriginal babies was more than double the − 135 g gap reported in 2015 Australian data [13]. Aboriginal status had no impact on birthweight when controlling for other pregnancy variables in a multivariable analysis (Table 2). The difference in birthweight in our study is explained by the relative prematurity of Aboriginal babies compared with non-Aboriginal babies. Further affecting this difference is the high number of complicated Aboriginal pregnancies transferred to our tertiary referral hospital. We acknowledge that being a tertiary hospital setting will result in a skewed representation of Aboriginal pregnancies compared with all possible Aboriginal pregnancies, but we expect our results would apply to Aboriginal pregnancies in similar urban settings to ours.

The Closing the Gap campaign has reported an improvement in rates of low birthweight babies born to Aboriginal compared with non-Aboriginal mothers, and cigarette smoking has been identified as a major contributor to the ongoing disparity [1]. Our results indicate that the higher rates of obesity and diabetes in Aboriginal mothers compared with non-Aboriginal mothers could be inflating birthweight and outweighing the negative effects of smoking. Consequently, although birthweight adjusted for gestational age was no longer lower in Aboriginal babies compared with non-Aboriginal babies, it does not mean that their health has improved, and all similar reports should be interpreted with caution.

International studies of Indigenous populations report increasing rates of obesity and diabetes which have led to more preterm but large for gestational age babies [14]. This result has been replicated in a study of Torres Strait Islanders, an Aboriginal sub-population known to have higher rates of obesity and diabetes, that had similar birthweight distributions uncorrected for gestational age when compared with the non-Indigenous population, but a greater risk of neonatal death [15]. These results suggest that preterm birth is a better predictor than birthweight of infant morbidity and mortality.

Our study found that 59.9% of Aboriginal women were overweight or obese, which was more than 42.6% of non-Aboriginal women at our hospital, and the rate of 46% for all Australian mothers who gave birth in 2015 [13]. Obesity in Aboriginal women has been proportionally linked to age and parity [16], and has been associated with increased risk of preterm birth due to comorbid hypertensive disorders and diabetes [17, 18]. More preterm birth was observed in our study with 23.3% of Aboriginal babies born before 37 weeks compared with 7.9% of non-Aboriginal babies. Prematurity has also been attributed to maternal smoking, teenage pregnancies, pre-eclampsia and drug abuse [19, 20].

The higher rates of diabetes, hypertensive disorders and pre-eclampsia found in Aboriginal pregnancies may be partially attributed to ethnic differences. Aboriginal female body types have a high degree of adiposity and central fat deposition [21], which predispose them to diabetes and hypertension [22]. Furthermore, Australian Aboriginals have been found to have an increased prevalence of a type 2 diabetes-susceptibility gene [23], which in combination with reported higher rates of obesity, physical inactivity and poor nutrition, put them at a much higher risk of cardiovascular comorbidities [24].

A similar retrospective cohort study was recently conducted in a non-tertiary Melbourne hospital, which concluded similar rates of low birthweight to Aboriginal and non-Aboriginal parents [25]. This discrepancy may firstly be due to differences in defining Aboriginal status, where maternal and paternal Aboriginal status was recorded on one database and the ethnicity of the baby was allocated if either parent was Aboriginal. No verification of Aboriginal status was performed through comparison with other administrative data sets and thus it was assumed that Aboriginal status was correctly recorded in all cases. Secondly, data was selected by birth episode and therefore did not acknowledge the high-risk pregnancies transferred in utero to tertiary centres. Our study shows significantly higher numbers of Aboriginal compared with non-Aboriginal pregnancies were transferred to our tertiary hospital with level 6 neonatal facilities (12.1% vs 3.5%), and these pregnancies were associated with lower birthweights (Table 2). This will skew data on Aboriginal birth outcomes in tertiary referral centres compared with regional centres and should be considered when interpreting conclusions.

### Limitations of the data

A major challenge of Aboriginal research and the development of effective policy is identifying Aboriginal people. Whilst recording of the mother’s Aboriginal status became mandatory in 1997 for the Perinatal National Minimum Data Set, it took until 2005 for all Australian jurisdictions to comply and until 2011 for the baby’s Aboriginal status to be added. Issues surrounding the identification of Australian Aboriginals include large fluctuations in numbers, the recording of a ‘not stated’ for Aboriginal status, hospitals not consistently asking patients of their Aboriginal status, and inconsistencies of Aboriginal classification with other records [26]. For these reasons it has been difficult to analyse the factors influencing Aboriginal health, compare data sets, and identify key areas for new policies.

Aboriginal status at RWH relied on self-reporting. Inspection of RWH data found 179 women who were identifying as Aboriginal that were in fact born overseas or spoke languages other than English or Aboriginal dialects at home. There were also 350 women who identified as Aboriginal at the time of birth but did not identify their newborn child as Aboriginal. Similar problems with Aboriginal data collection have been reported by the Australian Bureau of Statistics who found more people newly identifying as Aboriginal, the Aboriginal identification question being left unanswered, or people changing their Aboriginal status on official documents. Reasons for this are complex and may include fear of discrimination. Alternatively, it is possible that the term ‘Aboriginal’ may be misinterpreted, or there may be human error with selecting the correct Indigenous status. We achieved 97% concordance on Aboriginal status between our two administrative systems at RWH when only selecting mothers who identified both herself and her baby as being Aboriginal.

The retrospective manner of the study meant our data was reliant on self-reporting of drug, smoking and alcohol use, which is inherently likely to be under-reported. Verbal surveys of alcohol abuse in Aboriginals have been associated with under-reporting compared with non-Aboriginals, with females being more likely to under-report than males [27].

We acknowledge that the relatively small sample size of Aboriginal pregnancies limits the power to find small differences between groups.

## Conclusions

This study reports that Aboriginal women birthing in an urban area have higher rates of health risk behaviours and obstetric complications than non-Aboriginal mothers, and these are causing a birthweight disparity due to preterm delivery. There have been significant changes to Aboriginal lifestyles over time due to urbanization and government initiatives, but the subsequent effects on maternal wellbeing have not been adequately researched. The current study shows that maternal obesity, cigarette smoking and drug use are the main modifiable drivers of poor neonatal and obstetric outcomes in urban areas and these require targeted intervention programs. Aboriginal health can be further enhanced by refining administrative systems to identify Aboriginal pregnant mothers so that early referrals to support programs can be made and their outcomes appraised.

## Abbreviations

APH: Antepartum haemorrhage
BMI: Body mass index
CI: Confidence interval
GEEs: Generalised estimating equations
OR: Odds ratio
RWH: Royal Women’s Hospital
UTI: Urinary tract infection
